# *C^3^*: Consensus Cancer Driver Gene Caller

**DOI:** 10.1016/j.gpb.2018.10.004

**Published:** 2019-08-26

**Authors:** Chen-Yu Zhu, Chi Zhou, Yun-Qin Chen, Ai-Zong Shen, Zong-Ming Guo, Zhao-Yi Yang, Xiang-Yun Ye, Shen Qu, Jia Wei, Qi Liu

**Affiliations:** 1 Department of Endocrinology & Metabolism, Shanghai Tenth People's Hospital, Bioinformatics Department, School of Life Sciences and Technology, Tongji University, Shanghai 200092, China; 2 Department of Ophthalmology, Ninghai First Hospital, Ninghai 315600, China; 3 R&D Information, Innovation Center China, AstraZeneca, Shanghai 201203, China; 4 Shanghai Chest Hospital, Shanghai Jiaotong University, Shanghai 200240, China; 5 Department of Pharmacy, The First Affiliated Hospital of University of Science and Technology of China, Hefei 230036, China

**Keywords:** Somatic mutation, Cancer driver genes, Consensus, Data integration, Web server

## Abstract

Next-generation sequencing has allowed identification of millions of **somatic mutations** in human cancer cells. A key challenge in interpreting cancer genomes is to distinguish drivers of cancer development among available genetic mutations. To address this issue, we present the first web-based application, **consensus** cancer driver gene caller (*C^3^*), to identify the consensus driver genes using six different complementary strategies, *i.e.*, frequency-based, machine learning-based, functional bias-based, clustering-based, statistics model-based, and network-based strategies. This application allows users to specify customized operations when calling driver genes, and provides solid statistical evaluations and interpretable visualizations on the integration results. *C^3^* is implemented in Python and is freely available for public use at http://drivergene.rwebox.com/c3.

## Introduction

The continued advancement of next-generation sequencing (NGS) technology has allowed for the sequencing of large sets of cancer samples for somatic mutation discovery [1], [2]. However, one of the main challenges in interpreting the cancer genomes is to efficiently distinguish the driver mutations from the passenger mutations. Driver mutations are causally implicated in oncogenes and positively selected along the lineage of cancer development under the specific microenvironment conditions *in vivo*, whereas passenger mutations do not confer clonal growth advantages and are thus irrelevant to tumor development [3]. To address this issue, various methods have been proposed to identify driver genes based on distinctive assumptions and strategies [4], [5], [6], [7], [8], [9], [10], [11], [12], [13], [14], [15], [16]. Intuitively, all these driver gene identification strategies exhibit the biased signals of positive selection exploited by corresponding mechanisms at varied degrees. Several studies have been reported on benchmarking these methods with consensus cancer driver genes derived from individual model [8], [17], [18]. Collin et al. [8] proposed an evaluation framework to benchmark several existing models based on several measurements including precision, consistency, and mean log fold change (MLFC). Matan et al. [17] also benchmarked the available methods by using measurements such as precision and recall. Eduard et al. [18] classified four subtypes of driver gene calling methods at a subgene resolution. Denis et al. [19] provided the most comprehensive benchmarking of 21 driver gene prediction methods and proposed a Borda-based integration approach *ConsensusDriver*.

Despite these efforts, the available tools are often challenging for biologists or clinicians to carry out the related analysis directly, given the technical hurdles ranging from setting up the software to tuning parameters. A web-based user-friendly consensus driver gene prediction with intuitive visualization of the consensus mutation calling is needed. Here, we present the first web server-based consensus cancer driver gene caller (*C^3^*) platform to derive the consensus mutation calling results [4], [5], [6], [7], [8], [9], [10], [11], [12], [13], [14], [15], [16], [17], using six state-of-the-arts and complementary prediction strategies. These include frequency-based (*MutSigCV*) [6], machine learning-based (*20/20 +* ) [8], functional bias-based (*OncodriveFM*) [10], clustering-based (*OncodriveCLUST*) [11], statistics model-based (*DrGaP*) [5], and network-based (*MUFFINN*) [7]. Various calling evaluation and visualization strategies are incorporated in *C^3^* as follows. (1) *C^3^* provides a solid evaluation of the consensus mutation calling results with *Top-N-Precision* and *Top-N-nDCG* [20]. (2) *C^3^* provides an efficient integration strategy to derive the consensus results by Robust Rank Aggregation (*RRA*) [21] and statistical model-based intersection visualization [22]. (3) *Circos* plots are presented in *C^3^* to visualize the consensus mutation calling results [22], [23].

## Method

### General workflow of *C^3^*

**Figure 1:**
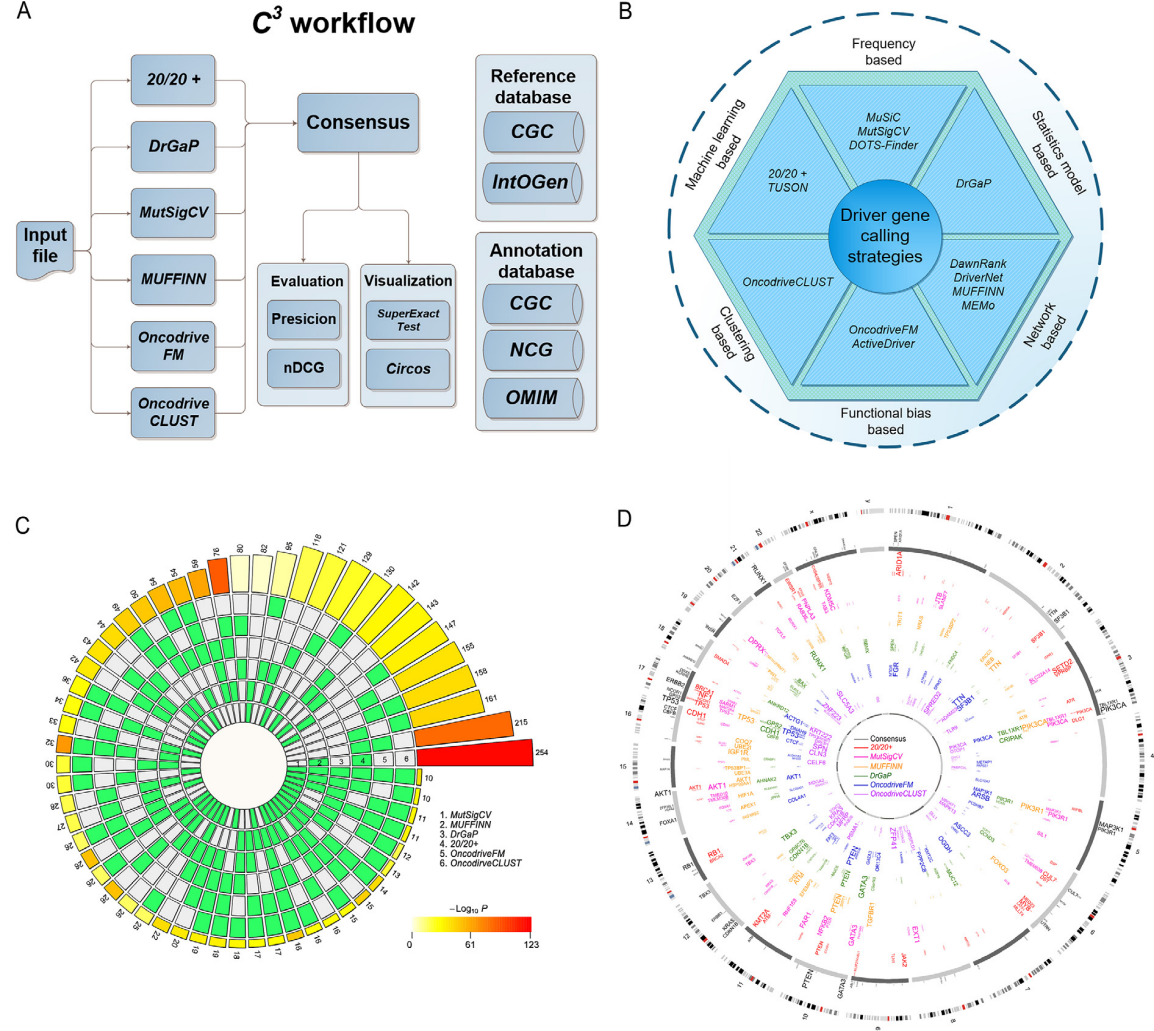
**Guideline of *C^3^* web server** **A.** A schematic representation of the *C^3^* workflow. A cancer sample input into *C^3^* workflow is analyzed by six cancer driver gene calling strategies (shown on the left), resulting in a consensus driver gene set as the output. Then the driver gene set is evaluated in terms of precision and nDCG before it is annotated based on the reference databases (shown on the right). Finally, the results are visualized by *SuperExactTest* and *Circos*. **B.** Overview of six categories of distinct cancer driver gene calling strategies. **C.***SuperExactTest* plot of the consensus calling results identified by *C^3^*. The inner rings (green and white blocks) represent six driver gene sets generated by the six strategies in *C^3^*. Blocks in white and green indicate the absence and presence of the driver gene sets, and each group represents an intersection of prediction results using 2–6 strategies (shown as the green blocks). The size of the intersections is proportionally shown by the heights of the bars on the outer ring. The number of each intersection is shown on the top of the respective bars and the color intensity of the bars represents the significance of the intersections (−Log_10_*P* value). **D.***Circos* plot of potential driver genes identified by *C^3^*. From the outer to inner circles, the first circle indicates the whole chromosomes across the genome and the second circle with gene symbols indicates the top 100 consensus driver genes identified by *C^3^*. The six inner colorful circles represent the top 100 results predicted by the individual strategies, respectively. Names of the strategies are provided in the center of the circle. The size of the gene symbol is positively proportional to the rank order of the predicted results, with a larger size indicating the higher rank. BRCA dataset in *TCGA* was used as an example for analyses shown in panels C and D.

*C^3^* accepts mutation annotation format (MAF) [24] file as input. The MAF file is annotated from variant calling format (VCF) [25] file, which can be acquired by using variant calling tool like *Mutect* on the NGS data. A schematic representation of the *C^3^* workflow is shown in Figure 1A. The selected programs, including *20/20+*, *MutSigCV*, *OncodriveFM*, *OncodriveCLUST*, *DrGaP*, and *MUFFINN* (Figure 1A and B; File S1 Part 1), run in the Ubuntu sever 16.04 system. Then all preprocessed input mutation data are processed in *C^3^* to obtain candidate driver genes list for each strategy separately. We use *SuperExactTest* model to evaluate the statistical significance of the intersection of individual calling results using all the protein-coding gene as a whole background gene set. In addition, based on each discrepant driver gene list, a rank ensemble method, *RobustRankAggreg*, is used to obtain a consensus driver gene list. Four databases including the Cancer Gene Census (*CGC*) [26], Integrative Onco Genomics (*IntOGen*) [10], Network of Cancer Genes (*NCG*) [27], and Online Mendelian Inheritance in Man (*OMIM*) [28] are used to annotate the predicted driver genes. Two evaluation measurements, *i.e.*, the *Top-N-Precision* and *Top-N-nDCG*, are applied to evaluate the calling performance. Finally, the *KEGG* [29] pathway and Gene Ontology analyses are also performed on the consensus driver genes for comprehensive annotations.

### Performance measurement

Previously, Collin et al. proposed a novel measurement of mean log fold change between the observed and desired theoretical *P* values [8]. Matan et al. [17] and Eduard et al. [18] applied measurements of precision and recall. Denis et al. also applied precision, recall, and F1 score [19] (File S1 Part 1). In our study, we applied the *Top-N-Precision* (using *CGC* data as a reference driver gene set [26]) and *Top-N-nDCG* (using *IntOGen* as a reference ranking driver gene set [30]) to facilitate the quantitative comparison and evaluation, focusing on the top *n* performance of the ranking results.

#### Precision

We evaluated the precision performance among the results acquired by the previous strategies based on the top 100 genes with respect to *CGC* cancer database through Equation (1). The average precision can measure a general predicting ability of individual methods among the pan-cancer cohort samples. We calculate the precision scores for each of 27 cancer types, and the SUM (precision) represents the sum of respective precision score of 27 cancer types (Equation (2)).

(1) $$\mathrm{Top-n-precision}=\frac{top\;n\;identified\;drvier\;genes\;overlapping\;with\;CGC}{top\;n\;identified\;driver\;genes}$$

(2) $$Average\;precision=\frac{SUM(precision\;of\;each\;individual\;cancer\;type)}{Number\;of\;cancer\;types}$$

#### nDCG

Meanwhile, normalized discounted cumulative gain (nDCG) was applied to measure the ranking quality of the results using the *IntOGen* as a reference cancer driver gene set.

(3) $$Weight\;of\;a\;reference\;gene=\frac{driver\;mutation\;counts\;in\;IntOGen}{SUM\;of\;drvier\;mutation\;counts\;in\;IntOGen}\times \left(No.\;of\;cancer\;driver\;genes\;in\;IntOGen-gene\;ranking\;by\;drvier\;mutation\;counts\right)$$

(4) $$\text{Weight of a gene}=\left\{\begin{array}{cc} 0 & \text{gene not available in the}\;IntOGen\;\text{dataset}\\ \text{Weight of a reference gene} & \text{gene available in the}\;IntOGen\;\text{dataset} \end{array}\right)$$

(5) $${CG}_{n}=SUM\left(weight\;of\;the\;top\;n\;predicted\;genes\right)$$

(6) $${DCG}_{n}={CG}_{1}+\sum_{i=2}^{n}\frac{{CG}_{n}}{{log}_{2}i}$$

(7) $${IDCG}_{n}={DCG}_{n(ranked\;by\;IntOGen)}$$

(8) $$\mathrm{Top-n-nDCG}=\frac{{DCG}_{n}}{{IDCG}_{n}}$$

(9) $$Average\;nDCG=\frac{SUM(nDCG\;of\;each\;individual\;cancer\;type)}{Number\;of\;cancer\;types}$$

Here, *n* represents the number of top predicted genes; *i* represents the rank of predicted genes; CG*_n_* represents cumulative weight of top *n* predicted genes; DCG*_n_* represents CG*_n_* multiplied by a discount factor $\frac{1}{\log_{2}i}$ (*i* > 1); IDCG*_n_* represents a DCG*_n_* under the ideal condition_,_ that is, the rank of predicted genes is exactly the same as that in the reference dataset. *Top-N-nDCG* represents normalized DCG*_n_* and measures the ranking performance of predicted genes.

To obtain the *Top-N*-*nDCG*, firstly, we download *IntOGen* cancer driver gene set (URL: https://www.intogen.org/) [31] and assign a weight for each reference driver gene in *IntOGen* based on their proportion of driver mutation counts [30] (Version 2014.12) calculated according to Equation (3). Specifically, the total number of cancer driver genes in *IntOGen* is 459. The weights of the predicted driver genes overlapping with the benchmark *IntOGen* dataset are calculated according to Equation (4). The weights of the predicted genes that are not available at the benchmark *IntOGen* dataset are set to 0. The *Top-N- nDCG* can be calculated through Equations (4), (5), (6), (7) [20].

### Rank aggregation

The *RRA* algorithm [21] is applied to obtain a consensus driver gene list, which aggregates the ranking driver genes predicted by individual tools. Comparing with the original *RankAggreg* algorithm [32], the *RRA* algorithm has three advantages: (1) it deals with incomplete rankings, which is common in practice, (2) it performs robustly with tolerance to the data noise, and (3) it is fast to be integrated for interactive data analysis.

### Intersection visualization and evaluation with *SuperExactTest* and *Circos*

We applied *SuperExactTest* [22] and *Circos* [23] to organize our visualization results. The former is a scalable visualization tool to illustrate high-order relationships among multi sets beyond Venn diagrams [33]. It evaluates the overlap of each of tools and presents a circular plot illustrating all possible intersections with statistical methods. The latter visualizes the predicted driver gene sets intuitively (Figure 1C and D; File S1 Part 5).

## Implementation

**Figure 2:**
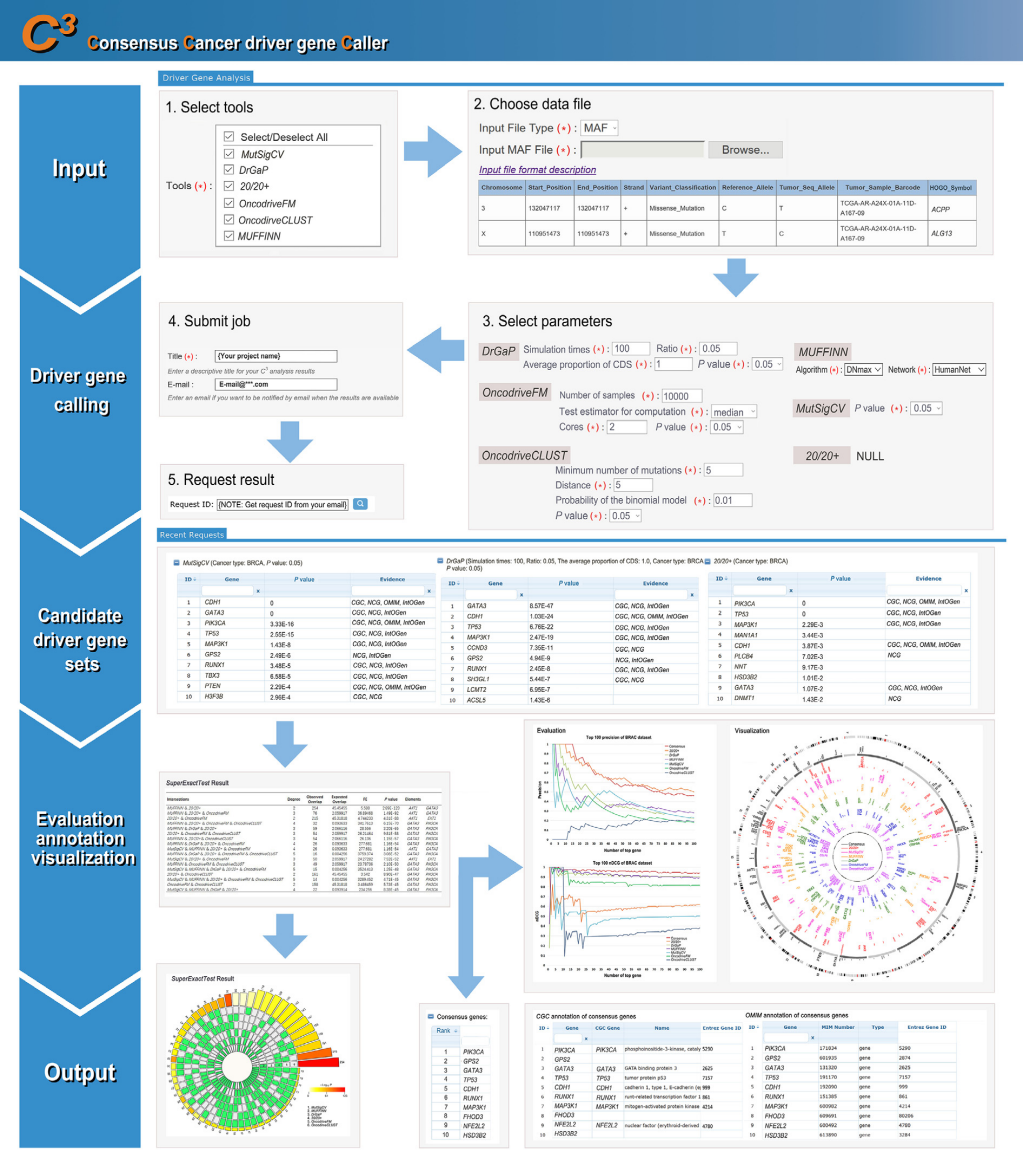
**General framework of *C^3^* web application** *C^3^* web application provides a user-friendly and simple five-step workflow. These include (1) selecting tools used for analysis, (2) choosing a data file to upload from user’s own computer (refer to our file format to verify the integrity of the input data), (3) selecting parameters for the selected tools (refer to the help documentation), (4) entering a name of the task (make sure to provide a valid e-mail address), and (5) inquiring and downloading the results with the request ID at “Recent Request” page. The request ID is sent to the user via e-mail upon the completion of the analysis.

As Figure 2 shows, *C^3^* web application accepts MAF [24] file or a modified micro-MAF file (Table S1) as the input. After users select driver gene calling strategies and parameters, *C^3^* runs as the back-end Ubuntu 16.04 system (with python-2.7, R-3.3.4 and *MATLAB Runtime 2014*). When the job is successfully finished, users will be notified through email including a “Request ID”. At the “Recent Request” page, users can preview and obtain candidate driver gene list by querying the “Request ID”. The output is directly viewable on the website and is available to downloaded for further analyses. The data submitted by every user are kept private. If there are any questions, users can visit the “Help” page for a detailed guidance.

### Detailed information of the test datasets

We test the stability of *C^3^* web application by selecting tumor datasets collected from The Cancer Genome Atlas (*TCGA*) [2] databases. Initially, the whole dataset includes 34 cancer types with 7724 samples and 729,235 mutations, curated from the published whole-exome sequencing or whole-genome sequencing studies which are also used by *TUSON* [9] and Collin study [8]. Since some tools (such as *MutSigCV* and *DrGaP*) need additional cohort mutation information, we removed 7 cancer types with 290 samples and 5164 mutations through data preprocessing. Finally, we curated 27 cancer types with 7434 samples and 724,071 mutations for the final analysis, which constitute the updated comprehensive test datasets finally for driver gene calling (Table 1 and File S1 Part 2).

**Table 1:** **Number of tested tumor samples and mutations**

**Cancer type**		**No. of samples**	**Total No. of mutations per cancer type**	**Average No. of mutations per sample**
**Abbreviation**	**Full name**			
BLCA	Urothelial bladder cancer	142	33,772	237.83
BRCA	Breast cancer	889	51,766	58.23
CESC	Cervical cancer	38	6115	160.92
CLL	chronic lymphocytic leukemia	224	3491	15.58
COAD	Colon adenocarcinoma	244	32,192	131.93
DLBCL	Diffuse large B-cell lymphoma	57	5785	101.49
ESCA	Esophageal cancer	160	19,141	119.63
GBM	Glioblastoma multiforme	365	21,923	60.06
HNSC	Head and neck squamous cell carcinoma	407	60,074	147.60
KIRC	Kidney renal clear cell carcinoma	484	28,483	58.85
KIRP	Kidney renal papillary cell carcinoma	112	7541	67.33
LAML	Acute Myeloid Leukemia	197	4180	21.22
LIHC	Liver hepatocellular carcinoma	151	7648	50.65
LGG	Lower Grade Glioma	227	9965	43.90
LUAD	Lung adenocarcinoma	394	106,613	270.59
LUSC	Lung squamous cell carcinoma	175	53,528	305.87
MB	Medulloblastoma	332	3615	10.89
MESO	Mesothelioma	289	97,806	338.43
MM	Multiple Myeloma	205	10,781	52.59
NBL	Neuroblastoma	352	6453	18.33
OV	Ovarian serous cystadenocarcinoma	480	28,136	58.62
PAAD	Pancreatic ductal adenocarcinoma	234	7939	33.93
PRAD	Prostate adenocarcinoma	420	16,784	39.96
STAD	Stomach adenocarcinoma	244	42,456	174.00
SCLC	Small cell lung cancer	31	8378	270.26
THCA	Papillary thyroid carcinoma	326	6424	19.71
UCEC	Uterine corpus endometrial carcinoma	255	39,234	153.86

### Performance of *C^3^*

**Figure 3:**
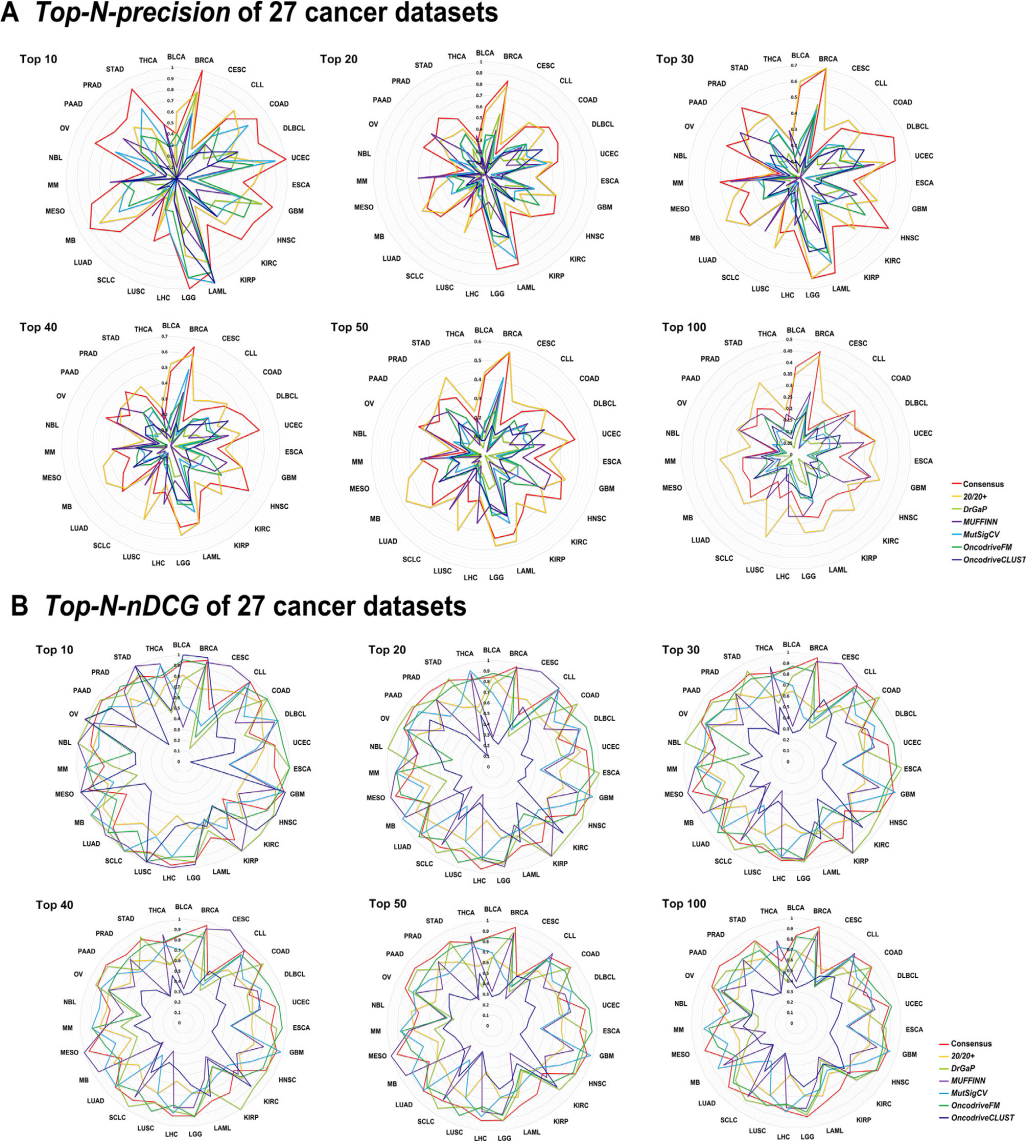
**Comparison of cancer driver gene calling performance using *Consensus* and the six individual strategies on 27 cancer datasets** The performance for *Consensus* and the six individual strategies on 27 cancer datasets is presented in radar plots in terms of the *Top-N-precision* (**A**) (calculated according to Equation (1)) and *Top-N-nDCG* (**B**) (calculated according to Equations (2), (3), (4), (5), (6)). Cancer types are labeled on the outmost circle. Values of precision in panel A and nDCG in panel B are labelled on each circle. The range of these values is between 0.1 and 1. For each cancer type, a higher value indicates a better performance and for each cancer driver gene calling strategy, the larger area means the better performance. nDCG, normalized discounted cumulative gain.

We benchmarked the performance of the consensus results comparing with each alternative. As shown in Figure 3, the integration results of *C^3^* application outperformed other methods evaluated with *Top-N*-*Precision* and *Top-N*-*nDCG*, revealing its superiority in driver genes prediction (File S1 Part 4).

*C^3^* also helps to identify reliable potential driver genes by *SuperExactTest* intersection between different driver gene calling strategies with reference to *CGC* and literature review. Detailed results are shown in Table S2 and Table S3.

In summary, although there exists a high discrepancy among different driver gene identification strategies, the intersection by individual strategies not only identifies the most reliable driver genes, but also helps to find potential novel driver genes that are not well-characterized.

## Future developments

Currently *C^3^* has some limitations and warrants future updates. (1) *C^3^* is currently deployed on the Ali Cloud server, which requires a lot of memory and space to process the data. Any variant file exceeding 40,000 records may fail when running *DrGaP*. Since the Random Forest Model *20/20 +* occupies too much CPU resources, it also takes a long time (>3 h for sample of 50,000 mutations with 8 cores of Intel Xeon E5-2643 3.3 GHz) to run a whole pipeline of *C^3^*. Future optimizations are required to accelerate *C^3^*. (2) Current version of *C^3^* only supports the GRCH37 reference genome, and a new version of the reference genome such as GRCH38 will be added in the next version. (3) One potential application of *C^3^* is to identify the target driver genes for drug discovery. However, the computationally predicted drivers should not be over-interpreted without additional experimental evidence.

## Availability

C3 is freely available for public use at http://drivergene.rwebox.com/c3.

## Authors’ contributions

QL, JW, XY, and SQ conceived the project. CYZ, CZ, YC, and ZG designed the platform. CYZ, AS, and ZY analyzed the data. QL, YC, CZ, and CYZ wrote the manuscript. All authors read and approved the final manuscript.

## Competing interests

The authors declare that they have no competing interests.

## Supplementary material

Supplementary File S1 **Detailed information of C^3^ application**
