# Supplementary material for: C3: Consensus Cancer Driver Gene Caller
Source: Genomics Proteomics Bioinformatics. 2019 Aug 26;17(3):311–8. doi: 10.1016/j.gpb.2018.10.004 (PMC6818389; doi:10.1016/j.gpb.2018.10.004)
Supplement: Supplementary File S1 — Detailed information of C3 application [file mmc1.docx]

**File S1 Detailed information of *C^3^* application**

**Part 1 Detailed information for individual cancer driver gene calling strategies**

Up to now there are more than 22,000 annotation genes [1], but the exact number of cancer driver genes is unknown. A clear definition to distinguish cancer driver genes from passenger genes has been lacking [2]. Various methods have been proposed to identify driver genes based on distinctive assumptions and strategies [3–15] (Table 1). In our study, we briefly categorized the existing methods into six different strategies. The basic strategy is to classify mutations based on recurrence, namely, the most commonly occurring mutations are more likely to be driver mutations [16] (frequency-based strategy). Several tools like *MuSiC* [3] and *MutSigCV* [5] identify genes that mutate more frequently than expected based on the background mutation rate (mutate recurrence) in a cohort. Therefore, the driver genes that mutate at a very low frequency are difficult to be detected using such approaches. Other strategies attempt to identify genes that exhibit other signals of positive selection across tumor samples, such as a bias towards the accumulation of functional mutations [9] (functional bias-based strategy), or the significant bias to cluster mutations in certain regions of the protein sequence [10] (clustering-based strategy). Furthermore, various features have been incorporated in statistical models to predict driver genes (statistical model-based strategy). Tools in this category include *DrGaP*, which integrates biological knowledge of the mutational process in tumors into the models. It extracts several features to infer the likelihood of a cancer gene to be a driver [4]. Meanwhile, machine learning-based methods have been proposed during recent years (machine learning-based strategy). *TUSON* [8] applies the LASSO model to predict the driver tumor suppressor genes (TSGs) and oncogene (OGs) based on somatic mutation information. Collin et al. [7] establish a formalized framework named *20/20+* (a random forest-based model) to evaluate the recurrent missense mutations and the proportion of inactivating mutations for driver gene identification. Last but not the least, network and pathway-based approaches are considered to offer a promising strategy for understanding driver genes due to their ability to model gene-gene interactions by aggregating an individual gene into a network or pathway (network-based strategy). Among them, *MUFFINN* identifies likely driver mutations by virtue of their pathway-centric method with high sensitivity [6].

**Table 1 Detailed information of pervious benchmark**

| **Author** | **Strategies involved** | **Measurements** | **Reference dataset** | **Ref.** |
| --- | --- | --- | --- | --- |
| Collin et al. | *MutSig suite*, *OncodriveFM*, *OncodriveFML*, *OncodriveCLUST*, *ActiveDriv*er, *MuSiC, TUSON*, *20/20+* | Precision  Consistency  mean log fold change (MLFC) | Cancer Gene Census | [7] |
| Matan et al. | *MutSigCV*, *OncodriveFM*, *OncodriveCLUST*, *ActiveDriv*er [14], *MuSiC*, *Gistic2.0*, *IntOGenCNV*, *Dendrix*, *HotNet2*, *Fusion-genes* | Precision  Recall | Cancer Gene Census | [17] |
| Eduard et al. | *Hotspot*, *NMC*, *OncodriveCLUST*, *MutSig-CL and iSIMPRe*, *iPAC*, *GraphPAC*, *SpacePAC and CLUMPS*, *e-Driver*, *ActiveDriver*, *LowMACA*, *e-Driver3D* | Precision  Recall | Cancer Gene Census | [18] |
| Denis et al. | *PolyPhen2*, *MutationTaster*, *MutationAssessor*, *SIFT*, *fathmm*, *CHASM*, *transFIC*, *AcveDriver*, *MutSigCV*, *OncodriveFM*, *OncodriveCLUST*, *Gistic2*, *MuSiC*, *S2N*, *OncodriveCIS*, *Conexic*, *NetBox*, *HotNet2*, *DriverNet*, *DawnRank*, *OncoIMPACT* | Precision  Recall  F1 score  Stability  Recovery | Cancer Gene Census | [19] |

**Part 2 Additional information of the test datasets**


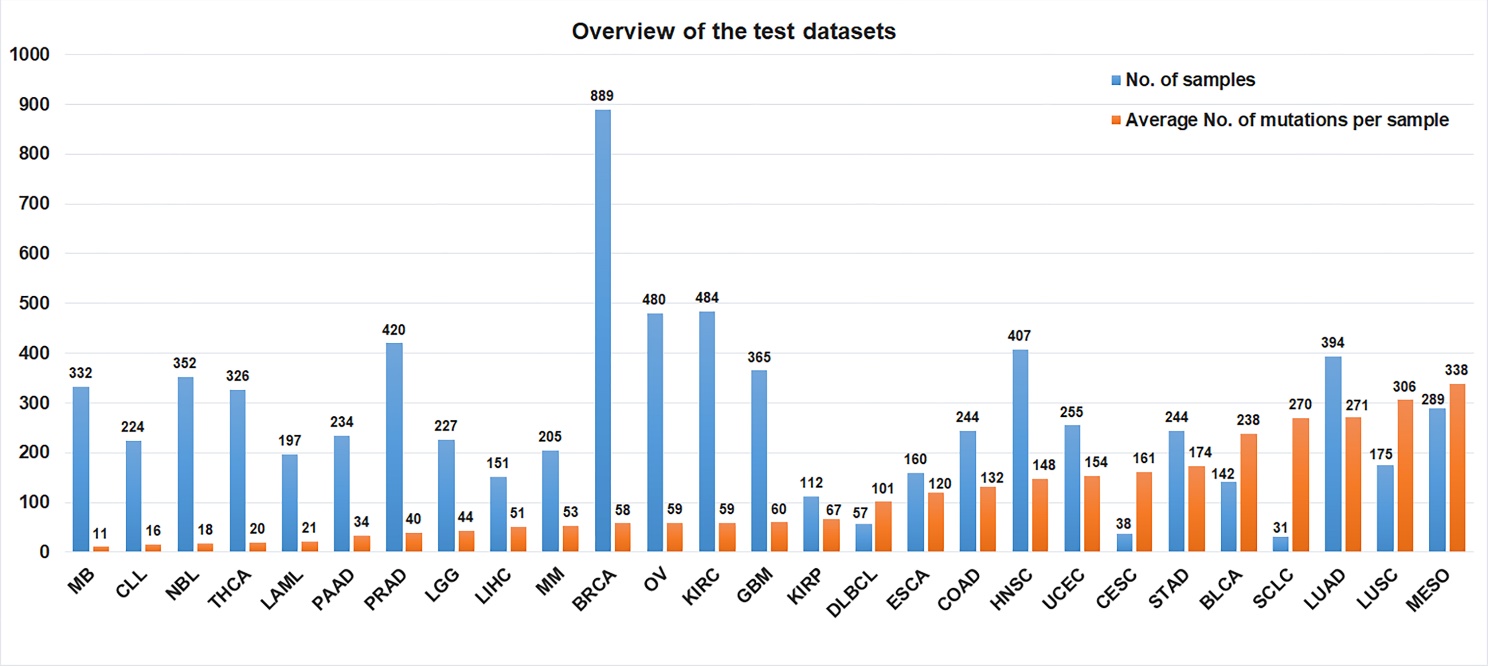


**Figure 1 Bar plot of the sample information of the test datasets**

27 cancer types with 7434 samples and 720,223 mutations were curated for the final analysis in our study. The blue bars show the number of samples in the respective datasets, ranging from 31 to 889. The orange bars show the average number of mutations per samples in the respective datasets, ranging from 11 to 338.

**Part 3 Detailed information of reference datasets**

To help evaluate the quality of the calling results, we obtained a list of 567 known driver genes from the well-studied cancer gene database, Cancer Gene Census (*CGC*) [20]. In addition, we also evaluated the rank results over the benchmark driver genes obtained from Integrative Onco Genomics (*IntOGen*) [9], which is a ranked driver gene list built on the well-known cohort studies over 27 tumor types. Furthermore, information from Online Mendelian Inheritance in Man (*OMIM*)[21] and Network of Cancer Genes (*NCG*) [20] is used to annotate the identified driver genes comprehensively (Table 2).

**Table 2 Description of benchmark gene sets and annotation databases**

| **Benchmark gene set** | **Abbreviation** | **No. of genes** | **Weblink** | **Ref.** |
| --- | --- | --- | --- | --- |
| Integrative Onco Genomics | *IntOGen* | 459 | https://www.intogen.org/search | [9] |
| Cancer Gene Census | *CGC* | 567 | https://cancer.sanger.ac.uk/census | [20] |
| Network of Cancer Genes | *NCG* | 1572 | http://ncg.kcl.ac.uk/ | [22] |
| Online Mendelian Inheritance in Man | *OMIM* | 348 | https://omim.org/ | [21] |

**Part 4 Benchmark of *C^3^* on 27 *TCGA* tested cancer types**

In this study, we applied two statistic measurements, *i.e.*, the *Top-N-Precision* using *CGC* as a reference driver gene set and the *Top-N-nDCG* using *IntOGen* [23] as a ranking reference cancer driver gene set, respectively, to evaluate the performance of *C^3^* and other individual strategies. *CGC* contains 567 essential cancer genes without ranking, thus *Top-N-Precision* is applied to reflect the prediction ability among the top *n* candidates without ranking. Since *IntOGen* contains the ranking reference driver genes according to the count of samples with percent accepted mutations (PAMs) [9]. *Top-N-nDCG*, a rank-based evaluation measurement, is applied to measure the ranking quality among the top *n* candidates in the cohort samples. In our study, *n* was empirically set to be the max value of 100 to investigate the performance of driver calling among the top 100 candidates. Based on the benchmark cancer driver genes contained in *CGC* and *IntOGen*, we proposed a rank aggregation-based model to derive the consensus driver gene calling results from six individual calling strategies (denoted as the *Consensus* in the following). As a result, Figure 2A depicted an average precision score on 27 tested cancer types. It can be seen that in general all the six individual strategies showed a declining tendency with *n* increasing, where precision was high at the start and then dropped nearly at the top 20. Among them, both *Consensus* and *20/20+* obtained a good performance, compared to others. *Consensus* also achieved a higher precision than *20/20+* before the top 37, indicating that *Consensus* optimizes the *Top-N-precision* than alternatives, and shows the potential to identify reliable driver genes among the most top rank candidates. From top 37 to 100, *20/20+* showed higher precision than *Consensus*. In Figure 2B, the average nDCG showed similar trends as observed for precision in Figure 2A. All tools achieved good prediction abilities to identify driver genes at top 10. After top 10, *Consensus* shows the best performance of nDCG among others.


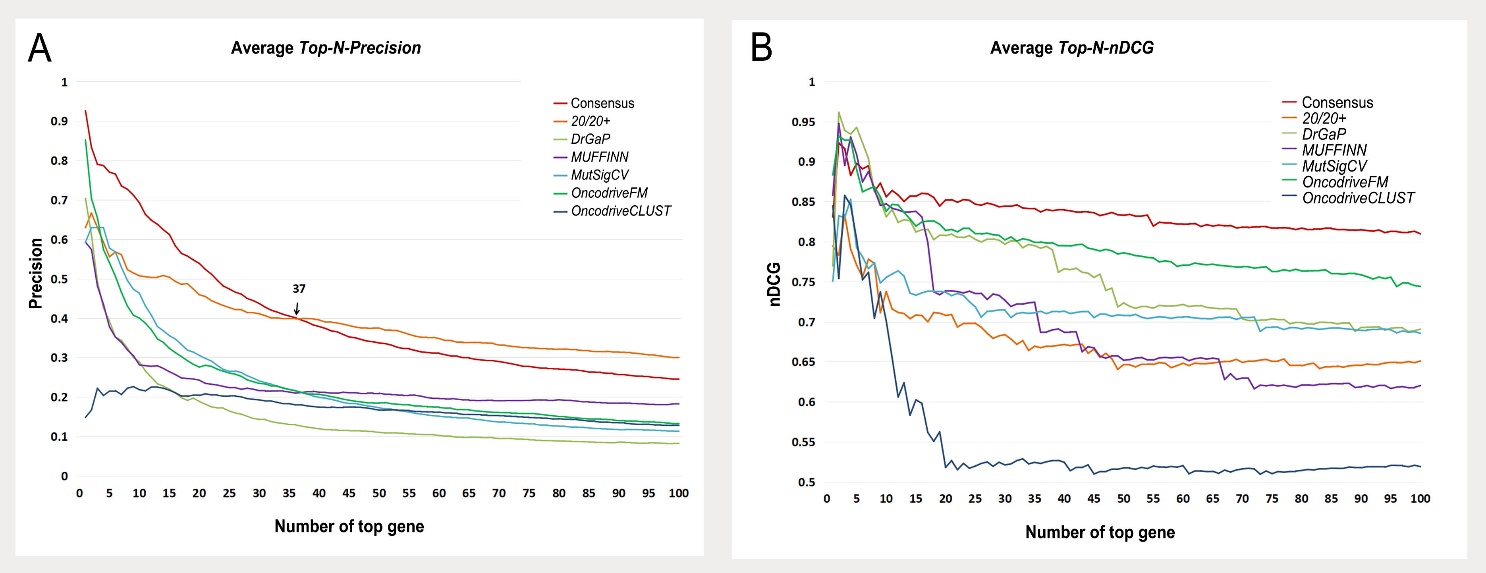


**Figure 2 Performance of top 100 candidates obtained by the *Consensus* and other strategies**

**A.** Averaged *Top-N-Precision* of *Consensus* and other strategies. **B.** Averaged *Top-N-nDCG* of *Consensus* and other strategies. BRCA, Breast cancer. nDCG, normalized discounted cumulative gain.

**Part 5 Consensus visualization**

*SuperExactTest*, a scalable visualization tool for illustrating high-order relationships among multi-sets beyond Venn diagrams [24], can evaluate the overlapping of each tool and show a circular plot illustrating all possible intersections and the corresponding statistics. Meanwhile, it provides us a novel approach for visualizing multi-set intersections (Figure 1C in the main text). Through such an intuitive visualization, users can easily identify the highest ranked genes. Further downstream annotations of these driver genes can be performed based on the information of *CGC* [20], *NCG* [22], and *OMIM* [21].

*C^3^* helps to identify reliable potential driver genes by *SuperExactTest* intersection among different driver gene calling strategies (See column “Confirmed genes from *CGC*” in Table S2). For all the 27 cancer types, the identified driver genes by *C^3^* had been confirmed with an average ratio of 60.58%, with reference to *CGC*. The rest of identified driver genes also show clearly literature evidence to be related to cancer development or cancer therapy (See column “Potential driver genes with literature evidence” in Table S2 and Table S3). These potential genes have accounted for 34.62% among all. In total, 95.2% of identified driver genes are either validated by *CGC* benchmark data or have clear literature evidence to support their relatedness to cancer mutagenesis.

**References**

[1] Tomczak K, Czerwinska P, Wiznerowicz M. The Cancer Genome Atlas (*TCGA*): an immeasurable source of knowledge. Contemp Oncol (Pozn) 2015;19:A68–77.

[2] Vogelstein B, Papadopoulos N, Velculescu VE, Zhou S, Diaz LA Jr, Kinzler KW. Cancer genome landscapes. Science 2013;339:1546–58.

[3] Dees ND, Zhang Q, Kandoth C, Wendl MC, Schierding W, Koboldt DC, et al. *MuSiC*: Identifying mutational significance in cancer genomes. Genome Res 2012;22:1589–98.

[4] Hua X, Xu H, Yang Y, Zhu J, Liu P, Lu Y. *DrGaP*: a powerful tool for identifying driver genes and pathways in cancer sequencing studies. Am J Hum Genet 2013;93:439–51.

[5] Lawrence MS, Stojanov P, Polak P, Kryukov GV, Cibulskis K, Sivachenko A, et al. Mutational heterogeneity in cancer and the search for new cancer-associated genes. Nature 2013;499:214–8.

[6] Cho A, Shim JE, Kim E, Supek F, Lehner B, Lee I. *MUFFINN*: cancer gene discovery via network analysis of somatic mutation data. Genome Biol 2016;17:129.

[7] Tokheim CJ, Papadopoulos N, Kinzler KW, Vogelstein B, Karchin R. Evaluating the evaluation of cancer driver genes. Proc Natl Acad Sci U S A 2016;113:14330–5.

[8] Davoli T, Xu AW, Mengwasser KE, Sack LM, Yoon JC, Park PJ, et al. Cumulative haploinsufficiency and triplosensitivity drive aneuploidy patterns and shape the cancer genome. Cell 2013;155:948–62.

[9] Gonzalez-Perez A, Lopez-Bigas N. Functional impact bias reveals cancer drivers. Nucleic Acids Res 2012;40:e169.

[10] Tamborero D, Gonzalez-Perez A, Lopez-Bigas N. *OncodriveCLUST*: exploiting the positional clustering of somatic mutations to identify cancer genes. Bioinformatics 2013;29:2238–44.

[11] Bashashati A, Haffari G, Ding J, Ha G, Lui K, Rosner J, et al. *DriverNet*: uncovering the impact of somatic driver mutations on transcriptional networks in cancer. Genome Biol 2012;13:R124.

[12] Hou JP, Ma J. DawnRank: discovering personalized driver genes in cancer. Genome Med 2014;6:56.

[13] Ciriello G, Cerami E, Sander C, Schultz N. Mutual exclusivity analysis identifies oncogenic network modules. Genome Res 2012;22:398–406.

[14] Reimand J, Bader GD. Systematic analysis of somatic mutations in phosphorylation signaling predicts novel cancer drivers. Mol Syst Biol 2013;9:637.

[15] Melloni GE, Ogier AG, de Pretis S, Mazzarella L, Pelizzola M, Pelicci PG, et al. *DOTS-Finder*: a comprehensive tool for assessing driver genes in cancer genomes. Genome Med 2014;6:44.

[16] Jones S, Zhang X, Parsons DW, Lin JC, Leary RJ, Angenendt P, et al. Core signaling pathways in human pancreatic cancers revealed by global genomic analyses. Science 2008;321:1801–6.

[17] Hofree M, Carter H, Kreisberg JF, Bandyopadhyay S, Mischel PS, Friend S, et al. Challenges in identifying cancer genes by analysis of exome sequencing data. Nat Commun 2016;7:12096.

[18] Porta-Pardo E, Kamburov A, Tamborero D, Pons T, Grases D, Valencia A, et al. Comparison of algorithms for the detection of cancer drivers at subgene resolution. Nat Methods 2017;14:782–8.

[19] Bertrand D, Drissler S, Chia BK, Koh JY, Li CH, Suphavilai C, et al. *ConsensusDriver* improves upon individual algorithms for predicting driver alterations in different cancer types and individual patients. Cancer Res 2018;78:290–301.

[20] Bamford S, Dawson E, Forbes S, Clements J, Pettett R, Dogan A, et al. The *COSMIC* (Catalogue of Somatic Mutations in Cancer) database and website. Br J Cancer 2004;91:355–8.

[21] Amberger JS, Bocchini CA, Schiettecatte F, Scott AF, Hamosh A. OMIM.org: Online Mendelian Inheritance in Man (*OMIM*(R)), an online catalog of human genes and genetic disorders. Nucleic Acids Res 2015;43:D789–98.

[22] Syed AS, D'Antonio M, Ciccarelli FD. Network of Cancer Genes: a web resource to analyze duplicability, orthology and network properties of cancer genes. Nucleic Acids Res 2009;38:D670–5.

[23] Rubio-Perez C, Tamborero D, Schroeder MP, Antolin AA, Deu-Pons J, Perez-Llamas C, et al. In silico prescription of anticancer drugs to cohorts of 28 tumor types reveals targeting opportunities. Cancer Cell 2015;27:382–96.

[24] Chen H, Boutros PC. *VennDiagram*: a package for the generation of highly-customizable Venn and Euler diagrams in R. BMC Bioinformatics 2011;12:35.
